# Association between flavonoid intake and arterial stiffness: PERSIAN cohort study

**DOI:** 10.22038/ajp.2025.25958

**Published:** 2025

**Authors:** Sara Ghodrat, Mostafa Shahraki Jazinaki, Reza Rezvani, Majid Khadem-Rezaiyan, Seyyed Mostafa Arabi, Saeid Eslami, Mohammad Hassan Abolhassani, Seyed Mohammad Tabatabaee Jabali, Abdolreza Norouzy

**Affiliations:** 1 *Department of Nutrition, Faculty of Medicine, Mashhad University of Medical Sciences, Mashhad, Iran*; 2 *Department of Community Medicine, Faculty of Medicine, Mashhad University of Medical Sciences, Mashhad, Iran*; 3 *Clinical nutrition and biochemistry department, Faculty of Medicine, Neyashbur University of Medical Sciences, Neyshabur, Iran*; 4 *Department of Medical Informatics, Faculty of Medicine, Mashhad University of Medical Sciences, Mashhad, Iran*; 5 *Department of Clinical Nutrition, Faculty of Medicine, Iran University of Medical Sciences, Tehran, Iran*; 6 *Department of Sports Medicine, School of Medicine, Iran University of Medical Sciences, Tehran, Iran*

**Keywords:** Flavonoids, Polyphenols, Arterial stiffness, Pulse wave velocity, Cardiovascular diseases

## Abstract

**Objective:**

Given the growing prevalence of cardiovascular diseases (CVDs), preventing them is considered one of the most critical global health goals. Evidence suggests that high flavonoid intake may reduce the risk of CVDs. Therefore, this study investigates the association between flavonoid intake and arterial stiffness as a predictor of CVDs.

**Materials and Methods:**

The PERSIAN cohort study in Mashhad data was used in this cross-sectional study. From data registered in the cohort center, 3423 men and women aged 35 to 70 years were recruited for this study. Total flavonoid intake and intakes of each flavonoid subclass were obtained through a food frequency questionnaire using the Phenol Explorer. Then, their association with blood pressure and arterial stiffness indices, including pulse wave velocity (PWV) and central augmentation index (AIx), was assessed using univariate logistic regression, and confounding factors were adjusted by performing the multivariable analysis.

**Results:**

The findings showed that total flavonoid intake had a non-significant inverse relationship with high-risk levels of PWV and AIx (OR (95% CI) for the highest quintile compared to the lowest quintile were 0.83 (0.65-1.06) and 0.95 (0.74-1.21), respectively). Also, no significant association was detected between intake of each flavonoid subclass and high-risk levels of blood pressure or arterial stiffness indices including PWV and AIx.

**Conclusion:**

This study revealed that total flavonoid and each flavonoid subclass had no significant association with high-risk arterial stiffness or blood pressure levels. More studies on flavonoids' impact on arterial stiffness are needed for a definite conclusion.

## Introduction

Flavonoids are a group of polyphenols that exist in many herbs, fruits, and vegetables (Tanwar and Modgil 2012). Flavonoids have a common basic chemical structure (Escobar-Cévoli et al. 2017). The structural components of the flavonoids include a basic flavonoid skeleton and a variation of multiple hydroxyl and methoxyl group substituents (Babu and Liu 2009). All of the main flavonoid subclasses are comprised of three six-membered rings: an aromatic A-ring fused to a heterocyclic C-ring attached through a single carbon-carbon bond to an aromatic B-ring. Flavonoids differ in the arrangements of hydroxyl, methoxyl, and glycosidic side groups and the conjugation between the A- and B- rings (Heim et al. 2002). These compounds are classified into six main subclasses based on structural differences, including anthocyanins, flavanols, flavonols, flavanones, flavones, and isoflavonoids (Brodowska 2017).

In recent studies, the relationship between flavonoid intake and the incidence of diseases such as cancer (Cutler et al. 2008; Kyle et al. 2010; Nimptsch et al. 2016), diabetes (Bondonno et al. 2021; Thompson et al. 2024), fatty liver (Bell et al. 2024), and cardiovascular diseases (CVDs) (Ponzo et al. 2015), has been highly regarded. 

An important feature of ageing is increased arterial stiffness, which various conditions and diseases, including atherosclerosis, chronic renal impairment, and diabetes, can associate with (Alinezhad‐Namaghi et al. 2023; Dumor et al. 2018; Stehouwer et al. 2008; Zanoli et al. 2019). Furthermore, a higher risk of CVDs, such as heart failure, myocardial infarction, and stroke, dementia, renal disease, and total mortality is associated with arterial stiffening (Stehouwer et al. 2008; Sutton-Tyrrell et al. 2005). Pulse wave velocity (PWV) is considered the gold standard for measuring arterial stiffness and is consistently predictive of CVD risk (Mitchell et al. 2010). In addition, the augmentation index (AIx) is a strong predictor of atherosclerosis (Chambless et al. 1997).

Although some interventional studies have examined the effect of the flavonoid-based intervention on PWV and AIx, the number of studies that investigated the association between dietary flavonoid intake and arterial stiffness is limited. Also, to the best of our knowledge, no large-scale study has yet investigated the relationship of total dietary flavonoid intake, considering various subclasses of flavonoids in addition to total flavonoid intake, with blood pressure and arterial stiffness. Therefore, the present study aimed to evaluate the association between total flavonoid and each flavonoid subclass intakes with blood pressure, and PWV and AIx as two indicators of arterial stiffness.

## Materials and Methods

### Participants and ethics

This cross-sectional study was approved by the Mashhad University of Medical Sciences, Mashhad, Iran, and Ethics Committee of the Medical School of Mashhad University of Medical Sciences with the number IR.MUMS.MEDICAL.REC.1399.406. An informed consent form was taken from all participants. The information required for this study was obtained from the Staff Health Monitoring Center. This information was collected by trained interviewers, general practitioners, nutritionists, and nurses. The inclusion criteria were being 35 to 70 years old, living in Mashhad, Iran, for at least one year, having Iranian citizenship, and completing the informed consent form. The exclusion criteria included pregnancy and lactation, diseases such as diabetes, cardiovascular disease, chronic kidney disease, cancer, and autoimmune diseases, consumption of supplements and medications that affect arterial stiffness, including antioxidant, vitamin D supplements, and medications such as glucocorticoids, antihypertensive drugs, and statins, calorie intake less than 800 or more than 4200 kcal and people whose information was incompletely recorded. Then, considering the inclusion and the exclusion criteria, among the 5622 participants in the PERSIAN Cohort of Mashhad, 3423 people were eligible to be included in the present study. 

### Data collection

The related data collected included general information, medical information, data from the food frequency questionnaire (FFQ), energy intake, macronutrients and micronutrients, level of physical activity, lifestyle, anthropometric data, arterial stiffness, and blood pressure from the PERSIAN Cohort database. The total flavonoid and each flavonoid subclass intakes were calculated using data collected from the FFQ based on the Phenol Explorer (PE) database. Then, the relationship between flavonoid intake and arterial stiffness indices, including PWV and AIx, and blood pressure (systolic blood pressure (SBP), and diastolic blood pressure (DBP)) was assessed.

### Anthropometric assessment

Individuals' body composition and weight assessments were performed using a bioelectrical impedance analyzer (BIA) named InBody 770 Biospace Co., Ltd., Seoul, Korea. All measurements were taken with minimal clothing and without shoes. All of the participants met the following criteria at the time of the BIA assessment: be well hydrated, have not exercised in the previous 4 to 6 hours, and have not consumed caffeine, alcohol, or diuretics in the last 24 hours (Mahan 2016).

Candidates were also asked to participate with an empty stomach and bladder. In addition, participants were asked not to have metal instruments in contact with the skin.

For height measurement, a digital height gauge (Inbody, BSM170 Biospace Co., Ltd. Seoul, Korea) with an accuracy of 1 mm was used. 

### Questionnaires

General information about individuals was collected using the PERSIAN Cohort general questionnaire. This questionnaire consists of 10 sections which include general characteristics (42 questions), economic and social status (27 questions), occupational status (7 questions), fuel status and place of residence (9 questions), lifestyle (13 questions), sleep (11 questions), physical activity questionnaire (28 questions), cell phone use (4 questions), pesticides (10 questions) and information about anthropometric examinations (6 questions). In the present study, information including age, gender, level of physical activity, and smoking was collected through this questionnaire and used for the present study.

Individual medical information was collected using the PERSIAN Cohort medical questionnaire. This questionnaire has eight main sections. These sections include fertility history (33 questions), chronic diseases (81 questions), medications (3 questions), family history of diseases (27 questions), oral health (9 questions), personal habits (4 questions), blood pressure measurement (11 questions) and physical examination (8 questions). The present study used information about pregnancy and lactation status, chronic diseases according to the exclusion criteria, and family history of CVDs and hypertension. 

The PERSIAN Cohort nutrition questionnaire was used to assess the dietary intake of individuals. This questionnaire includes four sections, including food frequency (112 questions), food supplements (10 questions), water consumption (2 questions), and eating habits (29 questions). The food frequency questionnaire, which has been used in many studies in Iran, is based on the Willet questionnaire and is designed by the Iranian food culture. It evaluates the food intake in the recent year (Ariya et al. 2020; Motamedi et al. 2021; Willett et al. 1985). For this purpose, individuals are asked to report the consumption of each questionnaire item by day, week, month, or year. This study used the food frequency questionnaire and information on supplement consumption. In addition, each food item's consumption in the questionnaire was provided in grams per day. In addition, information about calorie intake, macronutrients, and micronutrients calculated with version 7 of Nutritionist IV software was received from the Health Monitoring Center.

### Arterial stiffness assessment

Arterial stiffness and blood pressure were assessed using the SphygmoCor XCEL System (AtCor Medical, Australia). The Health Monitoring Center provided information for this study, including PWV, AIx, brachial systolic blood pressure (bSBP) and diastolic blood pressure (bDBP), central systolic blood pressure (cSBP), and central diastolic blood pressure (cDBP).

Arterial stiffness indices were assessed in the morning. Participants were examined when lying on their backs after at least 5 minutes of rest in a calm, temperature-balanced room. The arm cuff was first closed for the participant to assess systolic and diastolic brachial and central blood pressure and AIx. To assess carotid to femoral pulse wave velocity (cf-PWV), the femoral cuff was closed to create a femoral waveform, and the tonometer was placed at the carotid artery to create a carotid waveform. In addition, the distance from the carotid artery to the suprasternal notch and the distance from the femoral artery to the suprasternal notch was measured in millimeters. Then, the values ​​of each arterial stiffness and blood pressure indices are reported using Sphygmocor software.

### Flavonoid intake assessment

The studies mainly use the two databases, USDA (https://data.nal.usda.gov) and Phenol Explorer (PE) (http://phenol-explorer.eu), to assess flavonoid intake. This study searched food items from the food frequency questionnaire containing flavonoids in each database. The USDA database included about 78%, and the PE database included about 92% of the food items in the questionnaire. In addition, given that most studies have been using the PE database since 2012 to examine flavonoid intake due to its comprehensiveness, the research team has chosen this database to calculate flavonoid intake.

In the first step, the amount of flavonoids in each food frequency questionnaire food item was calculated based on the PE database. In this database, there were 89 types of anthocyanins, 45 types of flavanols, 49 types of flavones, 32 types of flavanones, 82 types of flavonols, and 92 types of isoflavones for different food items. Then, the total intake of flavonoid and its subclasses including flavanols, flavonols, flavanones, flavones, anthocyanins, and isoflavonoids was calculated. Some codes of food frequency questionnaire (FFQ) included several nutrients with different flavonoid content. To calculate the flavonoid content of some of these codes such as beans (code F2-1), mung bean/lentils (code F2-3), cabbage (code F5-2), green leafy vegetables (codes F5-5 and F5-6), raw celery/artichoke (code F5-8), beet/turnip (code F5-9), cherries / sour cherries (codes F6-5), peaches, nectarines, and berries (code F6-6), plums (code F6-10), grapes (code F6-12), juices (code F6-21), olive oil (code F7-5), and some nuts (code F7-10) and jams (Code F8-4), their average was considered. In addition, for some other codes, the flavonoid content of the most commonly consumed food was considered, such as green olives (code F7-6), green bell peppers (code F5-14), and white onions (code F5-13). 

In the second step, the dietary intake of each of the six flavonoid subclasses for each individual was calculated according to the amount of flavonoid content of the food items estimated in the first step. Finally, the total flavonoid intake of individuals was calculated from the sum of the intake of each of the six flavonoid subclasses.

### Statistical analysis

Statistical analysis was performed using SPSS software. In all analyses, p-value <0.05 was deemed as a statistical significance level.

Qualitative variables are described by frequency and frequency percentage. The normality of the quantitative data was checked using the Kolmogorov-Smirnov test. Quantitative variables with a normal distribution are presented as mean and standard deviation (SD), and quantitative data with a non-normal distribution are reported as median and interquartile range (IQR). Total flavonoids and flavonoid subclasses were divided into five equal groups based on the quintile. Also, individuals based on the measures of studied outcomes were divided into high-risk and low-risk groups based on the population means for PWV (7 m/s< high-risk) and Aix (26.7% < high-risk) or specific cut-off points for SBP (140 mmHg< high-risk) and DBP (90 mmHg< high-risk). Analysis was performed using univariate logistic regression by applying the enter method. An odds ratio with a 95% confidence interval is reported to compare the highest flavonoid intake with the first quintile for each outcome. 

Based on previous studies that reported the relationship between age (Lee and Oh 2010; Mitchell 2021), gender (Chen et al. 2023; DuPont et al. 2019; Wang et al. 2021), smoking (Gao et al. 2023; Hahad et al. 2023; Luehrs et al. 2021), BMI (Piko et al. 2023; Tang et al. 2020), level of physical activity (Park et al. 2017; Saz-Lara et al. 2021), and energy intake (Gómez-Sánchez et al. 2022; Redmond et al. 2010; Stanek et al. 2023), with arterial stiffness and blood pressure, these variables were identified as confounding factors.

Therefore, in the multivariable logistic regression model, the predictive ability of flavonoids for the studied outcomes in terms of age, gender, BMI, level of physical activity, and energy intake was adjusted.

## Results

A total of 3423 individuals, including 1506 men (44%) and 1917 women (56%), participated in the study. The baseline characteristics of the participants are presented in Table 1.

**Table 1:** Characteristics of participants (n=3423)

**Characteristic **	**Value** ^*^
Gender (Female, %)	56
Age (y)	43.7 ± 7.9
BMI (kg/m^2^)	26.3 ± 3.6
Physical activity (MET.h/day)	38.7 ± 5.3
Current smoker (yes, %)	6.8
FH of hypertension or CVDs (yes, %)	56.8
Energy intake (kcal/d)	2763.2 ± 675
Total flavonoid intake (mg/d)	752.4 ± 343
Flavanols (mg/d)	573.4 ± 300
Flavonols (mg/d)	107.8 ± 41
Flavones (mg/d)	36.3 ± 27
Flavanones (mg/d)	17.4 ± 12
Anthocyanins (mg/d)	13 ± 10
Isoflavonoids (mg/d)	4.2 ± 7
Systolic blood pressure (mmHg)	116.3 ± 12
Diastolic blood pressure (mmHg)	72.1 ± 8
Central systolic blood pressure (mmHg)	106.6 ± 11
Central diastolic blood pressure (mmHg)	72.9 ± 8
PWV (m/s)	7 ± 1
AIx (%)	26.7 ± 11

BMI, body mass index; FH, Family history; PWV, Pulse wave velocity; Aix, Augmentation index. *) Data is presented as mean + standard deviation or percentage.

**PWV and flavonoids**

The results of the crude model showed that the odds ratio (95% confidence interval) of high-risk levels of PWV for total flavonoid intake in the fifth quintile compared to the first quintile was 1.07 (95% CI: 0.87-1.33), which was not statistically significant. Also, in the second model, after adjusting in terms of age, gender, BMI, smoking, level of physical activity, and energy intake, this relationship remained non-significant (0.83 (95% CI: 0.65-1.06)).

In the crude model, flavanones and isoflavonoid intakes were inversely associated with high-risk PWV levels among flavonoid subclasses. However, this association was not statistically significant, and the OR (95% CI) for the fifth quintile compared to the first quintile of consuming these two types of flavonoids was 0.95 (0.77-1.17) and 0.83 (0.67-1.02), respectively. Furthermore, the adjusted model detected a non-significant inverse relationship between high-risk PWV levels and intake of all flavonoid subclasses except anthocyanins. These flavonoids can reduce the odds of high-risk levels of PWV by 6% (for flavanones) to 19% (for flavanols) (Table 2).

**Table 2:** Predictability of receiving the fifth quintile compared to the first quintile of total flavonoid and each flavonoid subclass intake on PWV based on binary logistic regression models and multivariable analysis (n=3423)

**Flavonoids**	**Crude model** **OR (95% CI)**	**Adjusted model** ^a^ **OR (95% CI)**
Flavanols	1.03 (0.83-1.28)	0.81 (0.64-1.04)
Flavonols	1.04 (0.33-1.49)	0.93 (0.72-1.19)
Flavones	1.01 (0.81-1.25)	0.93 (0.72-1.19)
Flavanones	0.95 (0.77-1.17)	0.94 (0.74-1.20)
Anthocyanins	1.26 (1.01-1.55)	1.15 (0.90-1.47)
Isoflavonoids	0.83 (0.67-1.02)	0.90 (0.70-1.16)
Total flavonoids	1.07 (0.87-1.33)	0.83 (0.65-1.06)

Abbreviations: OR: odds ratio, 95% CI: 95% confidence interval. OR was adjusted in terms of age, sex, BMI, smoking, level of physical activity, and energy intake

**AIx and flavonoids**

The results showed that total flavonoid intake was not significantly associated with high-risk levels of AIx. The OR (95% CI) of high-risk levels of AIx for the total flavonoid intake in the fifth quintile compared to the first quintile in the crude and adjusted models was 1.07 (95% CI: 0.86-1.33) and 0.95 (95% CI: 0.74-1.21), respectively.

The crude model demonstrated that the intake of isoflavonoids in the fifth quintile compared to the first quintile was significantly related to 34% lower odds of high-risk AIx levels. In addition, flavone intake in the fifth quintile was associated with 9% lower odds of high-risk AIx levels compared to the first quintile. However, it was not statistically significant (p = 0.43). In the adjusted model, the intake of all flavonoid subclasses except flavonols in the fifth quintile compared to the first quintile was inversely associated with 5-16% lower odds of high-risk AIx levels, non-significantly (p>0.05) (Table 3).

**Table 3:** Predictability of receiving the fifth quintile compared to the first quintile of total flavonoid and each flavonoid subclass intake on AIx based on binary logistic regression models and multivariable analysis (n=3423)

**Flavonoids**	**Crude model** **OR (95% CI)**	**Adjusted model ** **OR (95% CI)**
Flavanols	1.17 (0.95-1.46)	0.93 (0.73-1.18)
Flavonols	1.25 (1.01-1.55)	1.03 (0.81-1.31)
Flavones	0.91 (0.74-1.13)	0.84 (0.66-1.07)
Flavanones	1.08 (0.87-1.34)	0.90 (0.71-1.15)
Anthocyanins	1.00 (0.81-1.24)	0.85 (0.66-1.08)
Isoflavonoids	0.66 (0.53-0.82)	0.90 (0.71-1.14)
Total flavonoids	1.07 (0.86-1.33)	0.95 (0.74-1.21)

Abbreviations: OR: odds ratio, 95% CI: 95% confidence interval, SBP, systolic blood pressure; DBP, Diastolic blood pressure. OR was adjusted in terms of age, sex, BMI, smoking, level of physical activity, and energy intake

**Blood pressure indices and flavonoids**

The results showed a non-significant association between total flavonoid intake and high-risk blood pressure levels. In addition, total flavonoid intake in the fifth quintile compared to the first quintile was non-significantly associated with 20%, 3%, and 10% lower odds of high-risk levels of bDBP, cSBP, and cDBP, respectively. Also, there was a non-significant inverse association between the intake of all flavonoid subclasses except anthocyanin and high-risk levels of bSBP, bDBP, and cDBP. These flavonoid subclasses were non-significantly associated with 3-25% lower odds of high-risk levels of bSBP and bDBP and 4-11% lower odds of high-risk cDBP levels. Furthermore, intake of four flavonoid subtypes, including flavonols, flavones, flavanones, and isoflavonoids were non-significantly associated with 5-17% lower odds of high-risk cSBP levels (Table 4).

**Table 4:** Predictability of receiving the fifth quintile compared to the first quintile of total flavonoid and each flavonoid subclass intake on blood pressure indices based on an adjusted regression model (multivariable analysis) (n=3423)

**Flavonoids**	**bSBP** **OR (95% CI)**	**bDBP** **OR (95% CI)**	**cSBP** **OR (95% CI)**	**cDBP** **OR (95% CI)**
Flavanols	0.97 (0.69-1.37)	0.93 (0.60-1.45)	1.00 (0.78-1.27)	0.89 (0.70-1.12)
Flavonols	0.92 (0.66-1.28)	0.97 (0.63-1.49)	0.94 (0.74-1.19)	0.96 (0.76-1.21)
Flavones	0.83 (0.59-1.15)	0.85 (0.54-1.32)	0.88 (0.69-1.12)	0.86 (0.68-1.09)
Flavanones	0.91 (0.65-1.29)	0.92 (0.59-1.43)	0.95 (0.74-1.21)	0.98 (0.78-1.24)
Anthocyanins	1.03 (0.73-1.24)	1.04 (0.67-1.61)	1.03 (0.80-1.31)	1.01 (0.80-1.28)
Isoflavonoids	0.75 (0.54-1.06)	0.75 (0.49-1.17)	0.83 (0.65-1.06)	0.89 (0.71-1.13)
Total flavonoids	1.01 (0.72-1.42)	0.80 (0.51-1.26)	0.97 (0.76-1.23)	)0.900.71-1.14)

**Abbreviations:** OR: odds ratio, 95% CI: 95% confidence interval, SBP, systolic blood pressure; DBP, Diastolic blood pressure. OR was adjusted in terms of age, sex, BMI, smoking, level of physical activity, and energy intake

## Discussion

The present study found an inverse association between total flavonoid intake and high-risk PWV levels, although this association was not statistically significant. In addition, all flavonoid subclasses except anthocyanin were inversely associated with high-risk PWV levels, but this association was not statistically significant in any of the subclasses. Also, in the present study, it was observed that the total flavonoid and each of the flavonoid subclass intakes, did not have a significant association with high-risk Aix levels. In this regard, two studies have investigated the association between flavonoid intake and arterial stiffness indices. In the study conducted by Jennings et al., total flavonoid intake was not significantly associated with arterial stiffness indices, including PWV and AIx (Jennings et al. 2012). Therefore, their results are consistent with the findings of our study. However, contrary to our study's results, the amount of anthocyanins received in the fifth quintile compared to the first quintile was associated with a significant decrease in PWV. In this study, the average anthocyanin intake assessed by the United States Department of Agriculture (USDA) database was 17.7 ± 14.9 mg/day. Also, it has been shown that the highest anthocyanin intake was from red wine and berries. However, the mean ± SD intake of anthocyanin in our study was 13 ± 10.1 mg/day, as assessed using the PE database, and the highest amount of anthocyanin consumption was from cherries, sour cherries, and grapes, respectively. Therefore, the difference in the amount of anthocyanin intake, dietary sources, and the methods for flavonoid intake analysis could be considered determining factors to explain the difference in the results of these two studies regarding the association between anthocyanin intake and PWV. 

The meta-analysis conducted by Fairlie-Jones et al. showed that 1 to 8 hours after consuming anthocyanins, PWV levels significantly reduced, but AIx levels were not changed significantly. However, long-term use of this flavonoid did not significantly affect PWV or AIx (Fairlie-Jones et al. 2017). Considering the findings of this meta-analysis, the relationship between anthocyanin intake and arterial stiffness was different in the postprandial and long-term consumption.

In contrast to our findings, Jennings et al. reported that the amount of flavone intake in the fifth quintile compared to the first quintile was associated with a significant decrease in PWV. In this study, the amount of flavones intake was 2 ± 1.5 mg/day, and the most consumed dietary sources were citrus fruits, red wine, and peppers (Jennings et al. 2012). In contrast, in our study, the mean ± SD intake of flavones was 36.3 ± 27 mg/day, and the primary dietary sources were green leafy vegetables. 

Although there are limited observational studies on the association between flavonoid intake and arterial stiffness, many interventional studies have investigated the effect of different flavonoids on PWV and AIx. Most of these studies have examined the effect of isoflavonoids, flavonols, and anthocyanins as supplements or dietary sources on arterial stiffness. For example, a meta-analysis of randomized controlled clinical trials showed that consumption of soy isoflavonoids significantly reduces PWV and AIx. No significant difference was observed in this study between soy consumption of less than six months and six months and more than that duration or between different genders (Man et al. 2021). Furthermore, another meta-analysis study showed that consumption of cocoa products led to a significant decrease in PWV and AIx (Jafari Azad et al. 2021). 

Given the above evidence, it seems that although in the present study and some previous observational studies, there was no significant association between total flavonoid intake or most flavonoid subclasses and arterial indicators, the results of dietary interventions were promising. Various mechanisms have been proposed for the potential role of flavonoids in preventing increased arterial stiffness (Parmenter et al. 2020). For example, these compounds' antioxidant properties neutralize free radicals (Banjarnahor and Artanti 2014). Hydroxyl groups of flavonoids can react with and neutralize reactive oxygen species (ROS) (Kongpichitchoke et al. 2015). Furthermore, these hydroxyl groups also chelate metals such as iron and copper and prevent oxidative stress (Malešev and Kuntić 2007). In addition, flavonoids can reduce the activity of pro-oxidant enzymes such as inducible nitric oxide synthase (iNOS), NADPH oxidase, lipoxygenases, cyclooxygenase, xanthine oxidase, and mitochondrial succinate oxidase and instead activate antioxidant enzymes such as superoxide dismutase (SOD), glutathione peroxidase (GPx), and catalase (Banjarnahor and Artanti 2014; Malešev and Kuntić 2007; Procházková et al. 2011). Also, flavonoids increase the bioavailability of nitric oxide (NO), thereby preventing endothelial dysfunction (Qian et al. 2017). However, because arterial stiffness and CVDs occur gradually, short-term interventions may not considerably prevent the progression of arterial stiffness and CVDs. Therefore, it has been suggested that long-term dietary interventions may have a more significant impact on improving arterial stiffness and cardiovascular health. 

The results of the present study show that the intake of total flavonoids and flavonoid subtypes does not have a significant association with the high-risk levels of bSBP, bDBP, cSBP, and cDBP. The results of this study are in line with several previous studies. For example, a study by Jennings et al. showed that total flavonoid intake was not significantly associated with blood pressure parameters (Jennings et al. 2012). In this regard, a meta-analysis of 15 cross-sectional studies and 7 cohort studies showed that total flavonoid intake is not significantly associated with a reduction in the risk of hypertension. However, among the flavonoid subclasses, anthocyanin was significantly associated with an 8% reduction in the risk of hypertension (Godos et al. 2019). Although no significant relationship was observed between dietary flavonoid intake and blood pressure in our study, some mechanisms may lead to the hypotensive impact of flavonoid intake.

Flavonoids can effectively control hypertension through their antioxidant properties (Favari et al. 2020). Also, these compounds can reduce the risk of hypertension by affecting the regulatory pathways of blood pressure and regulating gene expression (Balasuriya and Rupasinghe 2011; Esser et al. 2018). In addition, flavonoids increase the production and bioavailability of NO which acts as a vasodilator (Duarte et al. 2014). Another possible mechanism for the effectiveness of flavonoids in lowering blood pressure could be the anti-inflammatory properties of these compounds (Ramdath et al. 2017). Evidence suggests that flavonoids exert anti-inflammatory effects by inhibiting NF-κB (Comalada et al. 2005). According to the mentioned mechanisms, many intervention studies have investigated the effect of different flavonoids on blood pressure indices. For example, one study found that consuming blackberry juice reduced endothelial dysfunction and blood pressure by lowering peroxy nitrate, a potent oxidant (Serraino et al. 2003). Among the dietary sources of anthocyanins, the significant reduction effects of pomegranate consumption on blood pressure have been reported (Bahari et al. 2024; Sahebkar et al. 2017), while no results have been shown for cherry (Eslami et al. 2022), strawberry (Shahraki Jazinaki et al. 2024), and raspberry intakes (Jazinaki et al. 2024). Among the dietary sources of isoflavones, a meta-analysis study shows that intake of soy protein in postmenopausal women lowers SBP and DBP levels. Also, this review reported that daily consumption of Soy isoflavone intake with a dosage of 100 mg ≤ led to a significant reduction in SBP and DBP (Kou et al. 2017). Among the dietary sources of flavanols, a meta-analysis that included 25 randomized controlled trials (RCTs) reported a significant reduction in blood pressure (SBP and DBP) followed by habitual tea consumption (Liu et al. 2014). Despite the existing studies, more studies are needed to investigate the long-term effects of total flavonoids and flavonoid subclasses consumption on blood pressure and arterial stiffness to draw a definite conclusion.

Due to the present study's cross-sectional design, it was impossible to investigate the causal relationship between the intake of flavonoids and blood pressure or arterial stiffness, which is the main limitation of this research. At the same time, this research has strong points such as high sample size, consideration of flavonoid subclasses in addition to the total dietary flavonoid intakes, and performing the multivariable analysis to adjust the confounding variables.

In conclusion, this study revealed that flavonoid subclasses and total flavonoid dietary intakes had no significant association with high-risk levels of arterial stiffness indices or blood pressure. However, to draw firm conclusions regarding this association, future RCTs should examine the impact of flavonoid supplementation or the consumption of flavonoid-rich sources on arterial stiffness indices and blood pressure.

## Data Availability

We don't have any research data outside the submitted manuscript file.
